# Draft Genome Sequence of *Pseudomonas stutzeri* Strain KMS 55, an Endophytic Diazotroph Isolated from Rice Roots

**DOI:** 10.1128/genomeA.00972-17

**Published:** 2017-10-05

**Authors:** Kannepalli Annapurna, Venkadasamy Govindasamy, Meenakshi Sharma, Yuvika Rajrana, Karivaradharajan Swarnalakshmi, Sangeeta Paul, Arpita Ghosh, Surendra K. Chikara

**Affiliations:** a Division of Microbiology, ICAR-Indian Agricultural Research Institute, New Delhi, India; b Eurofins Genomics India Private Limited, Bengaluru, Karnataka, India

## Abstract

*Pseudomonas stutzeri* strain KMS 55 (MTCC 12703) is an isolate from the root tissues of rice (*Oryza sativa* L.) that displays a high biological nitrogen fixation ability. Here, we report the complete genome sequence of this strain, which contains 4,637,820 bp, 4,289 protein-coding genes, 5,006 promoter sequences, 62 tRNAs, a single copy of 5S-16S-23S rRNA, and a genome average GC content of 51.18%. Analysis of the ~4.64-Mb genome sequence will give support to increased understanding of the genetic determinants of host range, endophytic colonization behavior, endophytic nitrogen fixation, and other plant-beneficial roles of *Pseudomonas stutzeri*.

## GENOME ANNOUNCEMENT

Beneficial endophytic interactions involve colonization of the inter- and intracellular spaces of plants by diverse groups of bacteria and fungi (1). The composition of the endophytic bacterial communities depends on the plant genotype, tissue, vegetation stage, and soil type (2, 3). Recently, we purified 110 bacterial isolates from the surface-sterilized root tissues of rice (*Oryza sativa* L.) genetic line no. 240 grown in the alluvial soil of the ICAR-IARI research farm, New Delhi. A Gram-negative, rod-shaped, motile, single-polar-flagellated, nonfluorescent bacterium was identified as a strain of *Pseudomonas stutzeri* based on 16S rRNA gene sequencing (GenBank accession number KY355732). The bacterium showed luxurious growth on N_2_-free mineral medium and nitrogenase activity in an acetylene reduction assay, and it is *nif* H positive. Also, *P. stutzeri* has been reported as a ubiquitous soil-dwelling, genetically diverse bacterium with denitrifying capabilities (4, 5). This isolate was named *Pseudomonas stutzeri* strain KMS 55 (MTCC 12703), and whole-genome sequencing was performed.

Genomic DNA from the exponential growth cultures (1 mL, optical density at 600 nm [OD_600_] 0.5) of *P. stutzeri* strain KMS 55 was extracted using the NucleoSpin DNA extraction kit (NucleoSpin, Germany). Genome sequencing was performed with paired-end sequencing libraries using a TruSeq standard Nano DNA library prep kit for Illumina at Eurofins Genomics India Pvt. Ltd. The Illumina library, with insert sizes ranging from 245 to 894 bp, was sequenced using 2 × 150-bp chemistry in a NextSeq-500. The high-quality paired-end short reads (2.6 Gb data of 8,994,174 reads) were assembled into scaffolds using SPAdes (version 3.7.1) (6). Gene prediction was performed with the help of Prodigal (version 2.60) using default parameters (7, 8).

In total, the assembly of 82 scaffolds resulted in a genome size of 4,637,820 bp with an average scaffold size of 56,558 bp and an *N*_50_ value of 399,608 bp with an average GC content of 51.18 mol%. A total of 4,289 genes in the range of 69 to 7,287 bp were predicted, with an average size of 963 bp. Furthermore, 67 RNA genes (3 rRNAs, 62 tRNAs, and 2 transfer-messenger RNAs [tmRNAs]) were predicted using RNAmmer (version 1.2) and the ARAGORN algorithm (9). Predicted genes were mapped onto the reference canonical pathways, followed by classification and functional annotation using the BLASTx program. Gene ontology distribution showed that among the 4,254 annotated genes, 1,797 genes were mapped to biological processes, 1,851 to molecular functions, and 1,248 to cellular components. Also, 39 nitrogen metabolism genes were annotated. Genes for nitrogen fixation and their regulatory elements, like *fix*L, *gln*K, *nif*A, and *rpo*N, were identified. A total of 5,006 promoter sequences were predicted, and a 53-bp clustered regularly interspaced short palindromic repeat (CRISPR) along with a 54-bp spacer sequence were also identified. This genomic information may be helpful in studying the genetic and functional characteristics of *P. stutzeri* strain KMS 55, a rice-colonizing endophytic diazotroph, and determining ways to improve its N-fixation potential through biotechnological interventions.

### Accession number(s).

The complete genome sequence of *Pseudomonas stutzeri* strain KMS 55 (MTCC 12703) has been deposited in DDBJ/ENA/GenBank under accession number MUEH00000000 (BioProject number PRJNA360478). The version described in this paper is the first version, MUEH01000000.
